# Contemporary Clinical Definitions, Differential Diagnosis, and Novel Predictive Tools for Renal Cell Carcinoma

**DOI:** 10.3390/biomedicines10112926

**Published:** 2022-11-14

**Authors:** Dorin Novacescu, Bogdan Ovidiu Feciche, Alin Adrian Cumpanas, Razvan Bardan, Andrei Valentin Rusmir, Yahya Almansour Bitar, Vlad Ilie Barbos, Talida Georgiana Cut, Marius Raica, Silviu Constantin Latcu

**Affiliations:** 1 Doctoral School, Victor Babes University of Medicine and Pharmacy Timisoara, E. Murgu Square, Nr. 2, 300041 Timisoara, Romania; 2 Department of Urology, “Pius Brinzeu” Timisoara County Emergency Hospital, Liviu Rebreanu Boulevard, Nr. 156, 300723 Timisoara, Romania; 3 Department of Urology, Satu-Mare County Emergency Hospital, Ravensburg Street, Nr. 2, 440192 Satu-Mare, Romania; 4 Department XV, Discipline of Urology, Victor Babes University of Medicine and Pharmacy Timisoara, E. Murgu Square, Nr. 2, 300041 Timisoara, Romania; 5 Department XIII, Discipline of Infectious Diseases, Victor Babes University of Medicine and Pharmacy Timisoara, E. Murgu Square, Nr. 2, 300041 Timisoara, Romania; 6 Center for Ethics in Human Genetic Identifications, Victor Babes University of Medicine and Pharmacy Timisoara, E. Murgu Square, Nr. 2, 300041 Timisoara, Romania; 7 Department II, Discipline of Histology, Victor Babes University of Medicine and Pharmacy Timisoara, E. Murgu Square, Nr. 2, 300041, Timisoara, Romania; 8 Angiogenesis Research Center, Victor Babes University of Medicine and Pharmacy Timisoara, E. Murgu Square, Nr. 2, 300041 Timisoara, Romania

**Keywords:** renal cell carcinoma (RCC), semiology, multimodal imaging, immunohistochemistry (IHC), molecular pathology, differential diagnosis, subtyping, prognosis, biomarkers, immunotherapy

## Abstract

Despite significant progress regarding clinical detection/imaging evaluation modalities and genetic/molecular characterization of pathogenesis, advanced renal cell carcinoma (RCC) remains an incurable disease and overall RCC mortality has been steadily rising for decades. Concomitantly, clinical definitions have been greatly nuanced and refined. RCCs are currently viewed as a heterogeneous series of cancers, with the same anatomical origin, but fundamentally different metabolisms and clinical behaviors. Thus, RCC pathological diagnosis/subtyping guidelines have become increasingly intricate and cumbersome, routinely requiring ancillary studies, mainly immunohistochemistry. Meanwhile, RCC-associated-antigen targeted systemic therapy has been greatly diversified and emerging, novel clinical applications for RCC immunotherapy have already reported significant survival benefits, at least in the adjuvant setting. Even so, systemically disseminated RCCs still associate very poor clinical outcomes, with currently available therapeutic modalities only being able to prolong survival. In lack of a definitive cure for advanced RCCs, integration of the amounting scientific knowledge regarding RCC pathogenesis into RCC clinical management has been paramount for improving patient outcomes. The current review aims to offer an integrative perspective regarding contemporary RCC clinical definitions, proper RCC clinical work-up at initial diagnosis (semiology and multimodal imaging), RCC pathological evaluation, differential diagnosis/subtyping protocols, and novel clinical tools for RCC screening, risk stratification and therapeutic response prediction.

## 1. Introduction

Over the past decades, major technological and scientific breakthroughs have allowed for the development of important clinical tools for better RCC detection, evaluation, and therapeutic decision-making, while also reshaping medical understanding of RCC pathogenesis and progression-driving molecularities. Even so, RCC remains, to this day, one of the deadliest urological malignancies, accounting for ~3% of the total cancer burden in the global adult population [1].

The widespread integration of ultrasonography (US) and computer tomography (CT) in routine clinical practice has significantly improved RCC detection yields, reflected in the long-standing and still ongoing stable increase in RCC incidence rates globally [2]. Concurrently, a significant drop in RCC initial stage at diagnosis has also occurred, with most RCCs being detected incidentally currently, as asymptomatic, localized, small renal masses. Within this subgroup of RCC cases, 5-year survival rates have been significantly improved as a consequence of earlier curative intervention, i.e., partial/radical nephrectomy [3]. Conversely, despite important recent progress in systemic RCC therapeutic management, advanced RCC patients remain incurable, with persistently poor clinical outcomes. Thus, overall, RCC-specific mortality rates have been steadily increasing since the 1990s (~1.1%/year) [3,4].

To address these pervasive clinical limitations, regarding systemic/recurrent RCC therapeutic management and outcomes, contemporary RCC clinical definitions have been greatly nuanced and refined to better serve in RCC subtyping, prognosis assessment, and therapeutic response prediction. Classically, RCCs were simply defined as malignant parenchymatous renal neoplasms, spawning from tubular epithelial cellularity. However, in light of recent extensive RCC genomic profiling efforts and comprehensive metabolic molecular analyses, RCCs are currently viewed as an extremely heterogenic group of distinctive tumor subtypes, which only share an anatomical origin, while having different cellular progenitors, within the renal parenchyma. In fact, individual RCC subtypes demonstrate relatively specific, yet widely pleomorphic, tumor-driving molecular pathologies and pathognomonic genomics. This emerging intricate molecular amalgamation of novel RCC metabolic profiles, is firmly corroborated by the similarly extensive and well-documented clinical variability in RCC malignant behavior and therapeutic response, both between different RCC cases and disease phenotypes comparatively, but also during the natural evolution of individual RCCs (disease progression, systemic dissemination, and/or metastatic recurrence) [5,6].

In lack of a definitive systemic treatment modality for advanced RCCs, integration of the amounting fundamental medical knowledge regarding RCC molecular pathology into RCC clinical management has been paramount to obtaining improved risk stratification and evidence-based therapeutic decision-making. As a result, definitive pathological diagnosis and RCC subtyping have become more nuanced and cumbersome, with the latest guidelines (i.e., the World Health Organization—WHO’s 4th edition of the Urological Tumors Classification, 2016) identifying almost twenty distinct subtypes of malignant renal cell tumors, alongside mesenchymal, neuroendocrine, nephroblastic, and cystic variants [5,7]. Even so, these classifications will only become more comprehensive further on, as additional RCC entities, with distinguishing clinical, morphological, immunohistochemical, and/or molecular traits, have already been identified and are still awaiting formal acknowledgement [8,9,10,11,12,13,14,15].

To further complicate the already elaborate and dynamic landscape of RCC clinical management, systemic RCC therapy has been greatly diversified recently, with the advent of personalized oncological therapy and RCC tumor microenvironment molecular/genomic characterization initiatives. Notably, focused evaluation of the biologic implications of various inflammatory pathways regarding RCC metabolomics and proliferation, mainly the Von Hippel–Lindau (VHL), mechanistic target of rapamycin (mTOR), tumor necrosis factor (TNF), and Janus kinase/signal transducer and activator of transcription (JAK/STAT) [16] pathways, has proven fruitful, providing multiple clinically relevant RCC-associated-antigen targeted molecules. Moreover, immunotherapy, a novel and promising type of oncotherapy, aiming to reactivate the cytotoxic immune response against tumor cells, has found important applications in advanced/recurrent RCC therapeutic management. Despite clinical difficulties regarding adequate immunotherapy response prediction for RCCs and the extensive therapy-associated costs, immunotherapy represents the only type of oncotherapy which allows, even theoretically, for the elaboration of a definitive cure for disseminated RCCs/cancer in general, as it is the only oncotherapy capable of targeting and annihilating non-dividing, quiescent, stem-like dysplastic cells [7,17].

With this acute need for harmonization in mind, between the effervescent field of RCC molecular pathology and the resulting best practice guidelines for RCC clinical management, we have elaborated the current literature review. The overarching aim is to facilitate RCC clinical management by offering a comprehensive review of contemporary RCC clinical definitions, semiology, and currently available imaging modalities for RCC detection and clinical work-up, while also focusing on RCC differential and definitive pathological diagnosis, subtyping, and prognosis assessment. Finally, we investigate and provide relevant data regarding emerging clinical tools for RCC screening, risk stratification, and therapeutic response prediction.

## 2. Semiology and Natural Evolution of RCCs

Anatomically, the kidneys are relatively isolated and inherently inaccessible, as there are paired organs located in the retroperitoneum. Thus, many renal tumors remain undiagnosed at an early stage and develop unencumbered, being asymptomatic and non-palpable. Currently, more than 60% of RRCs are detected incidentally, given the routine use of non-invasive imagining procedures, in the evaluation process of other non-specific symptoms or basic workup for other unrelated diseases [18,19,20]. This clinical development has increased the overall survival rate, at least in the case of organ-confined tumors [2,3,18,21].

During natural evolution (i.e., tumor progression and dissemination), RCC-related symptomatology occurs due to the following pathological mechanisms: local growth, hemorrhage, paraneoplastic syndromes, or metastatic disease [22]. If the tumor invades the collecting system and bleeding occurs, the resulting hematuria and/or renal colic due to ureteral blood clot obstruction, may represent, albeit rarely, an early alarm sign for organ-confined disease, or, more commonly, an indicator of locally advanced/invasive disease. Traditionally, a palpable abdominal mass, accompanied by gross hematuria and flank pain is known as the “late triad” (or Robson’s triad), indicating an advanced stage of the disease. This presentation is rarely seen today in clinical practice; yet, before the era of widely accessible imaging modalities, at the time of diagnosis, RCC patients would usually already manifest all or at least some of the late triad symptoms, indicative of a poor prognosis and an advanced/terminal stage of the disease. Other advanced disease signs/symptoms consist of unexplained weight loss, fever, night sweats, palpable cervical lymphadenopathy, unreducible varicocele, and bilateral edema of the lower extremity. A small subset of cases presents with symptoms associated with metastatic disease, such as bone pain or persistent cough. Spontaneous perirenal hemorrhage is a less frequent yet very important symptom of RCC. A study by Zhang et al. showed that for >50% of patients with unclear causes of perirenal hematoma, an asymptomatic renal tumor will be identified, most often AML or RCC [23,24]. Performing a CT after a few months helps establishing a definitive diagnosis.

Some of the rare clinical features of RCC are the paraneoplastic syndromes found in 10–20% of patients. Thus, RCC was previously considered the “internist’s tumor” due to the predominant systemic symptoms rather than local manifestations. Paraneoplastic syndromes rarely occur in small renal masses and are more common in end-stage disease [22,25]. Considering the increased incidental detection of RCC during medical imaging procedures, a more appropriate name would be the “radiologist’s tumor” [2,26].

It is important to identify paraneoplastic syndromes early on, as they can be a major source of morbidity and affect the decision-making process, during the management of patients with RCC. The most consistent clinical indicator, seen in >50% of RCC cases complicated with paraneoplastic syndromes, is an elevated erythrocyte sedimentation rate. Although non-specific, it may raise clinical suspicion and aid in early detection [27].

The kidneys are biologically active organs, regulating and maintaining systemic homeostasis, by producing multiple essential molecules involved in fundamental systemic processes, i.e., 1,25-dihydroxycholecalciferol, renin, erythropoietin, and various prostaglandins. In the presence of RCC, the kidneys produce a pathological amount of these molecular mediators, as well as additional, biologically active, de novo molecules, similar in structure to other innate human hormones, such as parathyroid hormone-like peptides, lupus-type anticoagulant, human chorionic gonadotropin, insulin, various cytokines, and inflammatory mediators. Collectively, these hormonal imbalances appear to be responsible for the development of paraneoplastic syndromes, alongside constitutional symptoms, i.e., weight loss. The RCC-derived hormonal alterations associate a range of endocrine dysregulations, with clinically significant systemic consequences: Cushing syndrome, hyperglycemia, galactorrhea, neuromyopathy, clotting disorders, and cerebellar ataxia [28]. 

In fact, as much as 13% of patients with RCC develop hypercalcemia due to either the paraneoplastic syndromes or an osteolytic metastasis of the skeletal system [29,30]. Hypercalcemia is usually suggested clinically by nausea, anorexia, chronic fatigue, and decreased deep tendon reflexes. The management involves efficient hydration followed by the administration of furosemide and the selective use of bisphosphonates, corticosteroids, or calcitonin. In fact, bisphosphonates are now considered the gold standard treatment for patients with malignant hypercalcemia, but only if the patient maintains an adequate renal function [30]. The administration of zoledronic acid 4mg intravenously every 4 weeks is effective in patients with RCC, in the presence of a normal renal function, and should be discontinued in case of renal insufficiency [30,31]. Alternatively, denosumab therapy can be considered in the second line, if hypercalcemia is refractory to bisphosphonates [22]. Based on the clinical circumstances, a more decisive management includes nephrectomy and occasional metastasectomy. Hypercalcemia due to RCC extensive osteolytic skeletal metastases is much more difficult to palliate, as it cannot be managed surgically. Nevertheless, many patients respond to bisphosphonate therapy and focused radiation therapy in cases with oligometastatic disease and a limited number of metastatic sites [31,32].

Other important paraneoplastic syndromes seen in RCC patients are hypertension and polycythemia [25,33]. Hypertension in RCC occurs due to an increase in production/secretion of renin, and/or by direct compression of the renal artery/distal branches (extrinsic compression or tumor invasion leading to renal artery stenosis), and/or secondary to arteriovenous fistula formation within the tumor. More uncommon causes of hypertension include polycythemia, hypercalcemia, obstructive uropathy with acute kidney injury, and an increased intracranial pressure secondary to possible cerebral metastases. RCC associated with polycythemia can be caused by the increased production of erythropoietin, either directly by the tumor or by the renal adjacent parenchyma, due to hypoxic events induced by tumor growth and progression [34].

Another paraneoplastic syndrome associated with RCC is non-metastatic hepatic dysfunction, also known as Stauffer syndrome, reported in 3–20% of cases [35,36]. Almost all patients affected by Stauffer syndrome have an increased serum alkaline phosphatase level, 67% have prolonged prothrombin time or hypoalbuminemia, and 20–30% have an increased serum bilirubin or transaminase levels. Thrombocytopenia and neutropenia are typical findings in Stauffer syndrome, as well as fever and weight loss. Due to a possible hepatic metastasis, a biopsy must be performed when imaging raises suspicion. Often, the hepatic biopsy will reveal a nonspecific hepatitis, associating regional necrosis. Stauffer syndrome usually entails increased serum levels of interleukin (IL)s, and it is believed that IL-6, among other cytokines, may play a pathogenic role. In 60–70% of patients with Stauffer syndrome at diagnosis, the hepatic function normalizes after radical nephrectomy. A persistence/recurrence of liver dysfunction is indicative of a pervasive viable tumor load, constituting a poor prognostic finding [35,36]. 

The optimal treatment of RCC-associated paraneoplastic syndromes involves surgical cytoreduction and/or systemic targeted oncological therapy, aiming to reduce tumor burden, if curability is no longer attainable. Except for the therapeutic management of hypercalcemia, other non-oncological medical therapies have not proved helpful.

## 3. The Role of Current Imaging Modalities in RCC Clinical Work-Up

The incidental detection of asymptomatic renal tumors has increased due to the inexpensive and widespread use of ultrasound (US) technology. The initial evaluation of parenchymal kidney lesions is usually done using B-mode US, which mainly distinguishes between cystic lesions and solid renal masses. For RCCs, gray scale US imaging shows an isoechoic or mildly hyperechoic, homogeneous or, especially for larger tumors, heterogeneous, solid parenchymal renal mass, with or without capsular bulging [37]. The additional use of color Doppler US allows for intra-tumor vascular distribution assessment and for additional quantification of RCC large vessel invasion, i.e., the detection of the characteristic RCC tumor thrombus extension, into the renal veins and/or inferior vena cava. Obviously, this is a crucial element in RCC clinical staging and for establishing the operative approach. The clinical suspicion of a RCC tumor thrombus, displayed on gray scale US as an echogenic body within the lumen of an enlarged vein, can be additionally supported by partial flow obstruction on Doppler US. Furthermore, the use of B-mode along with Doppler US is considered fairly accurate in establishing an initial differential diagnosis between a cystic and solid renal lesion, being a very useful alternative in case of limited access to superior cross-sectional imaging techniques, such as contrast-enhanced CT or magnetic resonance (MRI) [38]. However, complicated renal cysts still require a contrast-enhanced CT/MRI for final clinical diagnosis/staging.

In recent years, contrast-enhanced US (CEUS), a highly accurate, and, as compared to MRI/CT, very cost-effective diagnostic tool, has found increasingly wider applications in the clinical evaluation of RCCs. CEUS plays an important role in assessing renal lesions in patients with significant contraindications for the use of conventional contrast-enhanced imaging techniques, i.e., impaired renal function, hyperthyroidism or metallic implants. Since the contrast agent used in CEUS remains confined to the vascular system, the investigation has the major advantage of being usable regardless of renal and/or thyroid function. Moreover, it is very helpful in the case of patients who are allergic to gadolinium or iodine-based contrast agents [39,40,41]. Additionally, recent studies have reported the safe use of CEUS in pregnant women and children [42,43]. 

Regarding the clinical evaluation of RCCs using CEUS, it is essential to keep in mind that RCCs are intensely angiogenic tumors, demonstrating a high density of thin-walled blood vessels. This inherent conformation may facilitate the rapid circulation of CEUS contrast agents, while also possibly shortening the perfusion and clearance of set agents. Conversely, angiomyolipomas usually demonstrate irregular blood vessels, with narrow lumens, leading to slower circulation and longer perfusion and clearance of contrast agents. Thus, as a general principle, when using CEUS for the clinical evaluation of solid renal masses, malignant lesions display higher perfusion rates and shorter time-intensity curves than their benign counterparts. 

Notably, RCCs have a pronounced tendency to develop areas of necrosis, calcification, and/or active hemorrhage, and the absence of perfusion within these tumor areas may alter the reading of parameters during CEUS. Moreover, preexisting renal hypotrophy, especially when associating endophytic tumor growth, may impair RCC identification and lower CEUS image quality, due to the intrinsically greater distance from the body surface to the evaluated lesion, as compared to exophytic focal lesions in normotrophic kidneys. Even so, recently, the largest European investigation focusing on the ability of CEUS to predict malignancy within the clinical evaluation of renal lesions, using histological validation, reported a high effectiveness and predictive accuracy for CEUS, comparable to, if not greater than those of more expensive imaging modalities [44]. Beyond its diagnostic usefulness in RCCs, CEUS can also be helpful during RCC therapeutic procedures, such as percutaneous radiofrequency ablation, where it may be used as a real time monitoring technique, without affecting glomerular filtration rate levels [45].

Moving on to the more modern, multiphasic, cross-sectional, contrast-enhanced, imaging techniques—CT, MRI, molecular positron-emission tomography (PET-CT)—we most note that, even though these tools have greatly improved both the sensitivity of RCC detection/initial diagnosis, as well as clinical staging abilities (i.e., loco-regional extension and systemic dissemination) for overtly malignant lesions, little progress has been made regarding the specificity of primary renal malignancy differential diagnosis through non-invasive imaging modalities. Aside from the Bosniak classification system for cystic renal lesions [46] and composite complexity profiles (the R.E.N.A.L. nephrometry score, the PADUA score, and the C-index [47,48,49]) used for assessing primary renal tumor complexity, therapeutic decision-making (radical nephrectomy vs. nephron-sparing surgery/open vs. minimally invasive), and surgical complications risk assessment [50,51,52], no specific, formal, imaging protocol has yet to be elaborated for differentiating between benign and malignant solid renal masses, nor between RCC subtypes.

Due to the significant overlap of cross-sectional imaging characteristics for oncocytoma, clear cell RCC, and papillary RCC subtypes [53,54], a complete and definitive characterization of RCC’s biological potential cannot be obtained solely based on this method. Thus, a meticulous pathological evaluation of tumor specimens must be undergone for definitive diagnosis, prognosis, and personalized therapeutic management. However, with the limitation of baseline patient renal function (risk of post-contrast nephropathy), the differential diagnosis of solid renal masses must consider a few validated imaging principles, namely:

- infiltrative growth pattern of solid renal masses, although suggestive for malignancy, is not pathognomonic for RCC, only broadening the differential diagnosis (urothelial carcinoma, renal lymphoma, renal abscess, and high-grade or sarcomatoid RCC);
- enhancement of ˃15–20 Hounsfield units (HU) on CT is indicative of RCC, without excluding benign histology [55];
- large areas with negative CT attenuation numbers (<−20 HU) are indicative of intralesional fat and diagnostic for angiomyolipoma (AML) [56,57];
- enhancement of >20% with intravenous gadolinium-based contrast on MRI is suggestive of RCC (especially helpful in masses ˂2 cm) [58];
- papillary RCCs are often hypo-enhancing relative to other subtypes of RCC [54,59].

Novel molecular imaging tracers, i.e., high-affinity, radio-labeled, targeted molecules, aim to exploit and quantify known, high-specificity, key cellular processes involved in malignant tumor biology, in order to improve diagnostic imaging. Fluorodeoxyglucose (FDG), the most widely available tracer for PET-CT, has a very limited role in RCC differential diagnosis, due to its renal excretion and implicit high levels of visual conspicuity in the kidney and at the most common sites of metastatic disease [60]. Furthermore, 99mTc-sestamibi single-photon emission CT (SPECT) has demonstrated promising initial results in distinguishing renal oncocytoma and hybrid oncocytic/chromophobe tumors from clear cell RCC, via mitochondrial uptake documentation (abundant in oncocytic and chromophobe tumors), and is awaiting largescale validation [61]. Other RCC applications for novel tracers remain in developmental stages, but may prove useful in the future: 124I-girentuximab, targeting carbonic anhydrase (CA) IX (suggestive for clear cell RCC) [62], and prostate-specific membrane antigen (PSMA), with its epithelial membrane target [63]. Even so, these diagnostic tools are quite far from being available for clinical practice.

## 4. Definitive Diagnosis in RCCs: Pathology, Genetics, and Ancillary Studies

Currently, as clinical definitions have become greatly nuanced, a complete RCC diagnosis requires careful multimodal evaluation, in a well-coordinated and phased approach, involving, at times, very difficult differentials.

### 4.1. Conventional Staining amd RCC Microscopic Morphological Evaluation

Notwithstanding the long-held, central role of RCC morphological evaluation, using standard microscopy and conventional hematoxylin–eosin (HE) staining, in RCC definitive pathological diagnosis, essential for further therapeutic decision-making and predicting prognosis/risk stratification, in light of recent insights into RCC molecular pathology, the method, although still important, has become insufficient for adequate RCC subtyping. Even within the classical triad of conventional morphologies, namely clear cell (ccRCC), papillary (pRCC), and chromophobe (chRCC), which demonstrate relatively distinctive architectures and cellular features, microscopic evaluation of morphology remains subjective and user-dependent. Additionally, more often than not, meticulous evaluation will encounter, at least focally, overlapping patterns and/or atypical architectures, hindering definitive RCC subtyping. High-grade cellularity and severely dedifferentiated renal tumors lose characteristic morphological traits completely and cannot be subtyped using solely conventional staining microscopy. Determining metastatic cell origin, in advanced RCCs, with the additional difficulty of non-renal origin differentials, will most certainly require ancillary studies [6]. 

In fact, as a general consensus among pathologists, no truly reliable, objective, and validated histologic/ultrastructural criteria exist for distinguishing between benign and malignant renal epithelial tumors [30], with the very meek exception of oncocytomas and small low-grade papillary adenomas (≤5 mm) [30]. Furthermore, the amounting, emergent RCC clinical entities, have shown, for the most part, unspecific and poorly defined architectures, with heavily disputed morphological growth characteristics. Even within conventional RCC morphologies, absolute homogeneity is extremely rare and non-specific arrangement patterns (solid, papillary, tubular/cystic, sarcomatoid/rhabdoid) and cellular features (cytoplasmic clearing, eosinophilia/basophilia) are routinely identified, particularly in high-grade and/or poorly preserved tumor areas. Clearly, meticulous sampling of gross RCC specimens, especially in those which prove to be difficult to classify, will prove to be much more useful and cost-effective than additional staining. Transitional areas, from well-differentiated/low-grade patterns to more unusual, pleomorphic patterns, should be sought after and evaluated preferentially, as they usually offer the most useful diagnostic information [6,64].

All in all, conventional microscopic evaluation of RCC morphology remains an important initial step in tumor pathological analysis. It allows for an essential primary classification into RCC predominant morphological trait subgroups, simplifying the differential diagnosis and guiding further targeted analysis, while greatly reducing financial costs. Even so, it has become insufficient for an accurate definitive RCC diagnosis and prognosis assessment.

### 4.2. Definitions and Comprehensive Profiles for RCC Subtyping

Definitive RCC pathological diagnosis, meaning irrefutable demonstration of tumor renal cell origin and accurate RCC subtype identification, as well as the subsequent prognosis assessment, requires careful integration of clinical, pathological, and molecular characteristics, to allow for objective carcinogenic metabolic profiling and characterization of progression-driving tumor biology. Thus, as our understanding of RCC molecular pathology has become more nuanced, ancillary studies, mainly immunohistochemistry (IHC), has become integrated in clinical practice, as essential tools for routine RCC pathological evaluation. Despite the plethora of seemingly promising diagnostic and predictive applications reported, for the dozens of novel tumor-associated molecules and their corresponding IHC targeted-antibodies, due to the lack of validation studies, these data constitute no more than level 2/3 evidence. Moreover, heterogeneity within existing data regarding specific IHC methodology and antibody clone used (monoclonal/polyclonal) further encumbers comparisons between existing results [65,66,67,68,69,70,71]. In fact, as an investigative method, IHC has multiple inherent conceptual limitations, as well as significant technical (clone selection, titration, validation, false positives/negatives etc.) and interpretative (subcellular localization and pattern) weaknesses [64]. We provide a comprehensive summary of evidence regarding morphological, IHC, and genetic profiles for all currently accepted RCC subtypes in Table 1.

A recurring issue is represented by the indiscriminate use of an inordinate number of antibodies, without a reasonable, structured, diagnostic approach, which only serves to generate additional confusion, while simultaneously wasting valuable resources. For this reason, in 2012, the International Society of Urological Pathology (ISUP) reviewed the use of IHC antibodies in adult renal tumors, in order to develop best practice recommendations regarding determining site of origin, typing, and prognosis, with the ultimate goal of establishing formal panels of biomarkers for specific diagnostic difficulties and, implicitly, establishing adequate guidelines for stewardship [6,64]. For a more systematic and practical approach to RCC subtyping, individual, differential diagnosis-driven IHC marker panels have been established (see Table 2), in order to differentiate among entities within the main RCC subgroups, manifesting specific morphological characteristics, namely predominantly clear cell (cc) population, significant papillary (p) component, extensive cytoplasmic eosinophilia, predominant sarcomatoid growth pattern, and architecture suggestive of distal nephron origin—collecting duct carcinoma (CDC) and renal medullary carcinoma (RMC). Quantitative and qualitative assessments of the staining results are equally important and continuous refinement of antibody panels, taking into account the proven value of new IHC markers and new clones of established markers, as they enter the market, is mandatory [6].

Overall, Paired box (PAX) 8, a transcription factor (415 aminoacids/48 kDa), involved in kidney, thyroid, and paramesonephric duct-derived, organogenesis, and homeostasis, represents the most useful IHC marker for establishing the diagnosis of metastatic RCC(mRCC) [72], being expressed in all RCC subtypes, including sarcomatoid RCC, mucinous tubular and spindle cell carcinoma (MTSC), and microphtalmia (MiT)-family translocation RCC, with a sensitivity of approximately 95% [73]. In healthy kidney tissue, PAX8 normally manifests diffuse staining in the, preferentially distal, renal tubules, and patchy staining of urothelium in the renal pelvis. In accordance with this pattern of developmental expression, in addition to RCCs, PAX8 consistently stains Műllerian neoplasms and thyroid neoplasms, and, in smaller subsets (~20%), urothelial carcinomas of the renal pelvis, Wolffian duct lesions, and thymic neoplasms [6].

Out of the PAX gene family for tissue specific transcription factors [74], PAX8 is generally the more sensitive marker [75]. PAX2 stains similarly to PAX8 [76,77]; with the possibly useful difference of negative PAX2 staining in thyroid neoplasms, admittedly only reported in small series [78]. When using older, polyclonal preparations, endocrine neoplasms (pancreatic islet cell tumors and gastrointestinal tract carcinoids) are often positive for PAX8; however, cross-reactivity with PAX6 is clarifying [79]. Additionally, B-cell lymphomas stain positive on polyclonal PAX8 preparation, while also manifesting cross-reaction with PAX5.

**Table 1:** Summary of evidence regarding RCC histological, IHC, and genetic profiles [5,6,64,80,81,82,83,84,85,86,87,88,89,90,91,92,93,94,95,96,97,98,99,100,101,102,103,104,105,106,107,108,109,110,111,112,113,114,115,116,117,118,119,120,121,122,123,124,125,126,127,128,129,130,131,132,133,134,135,136,137,138,139,140,141,142,143,144,145,146,147,148,149,150,151,152,153,154,155,156,157,158,159,160,161].

TYPE	MYCROSCOPY	IHC STAINING	GENETICS
**Clear cell (cc) RCC**	**Hypercellularity**: nests/sheets of cells, manifesting cytoplasmatic clearing and individualized membranes. Granular eosinophilic cytoplasm, seen in high-grade areas or near hemorrhage/necrosis. Rarely, intra/extracellular hyaline globules, basophilic cytoplasmic inclusions, abundant multinucleated giant cells. **Architecture**: solid, alveolar/nested, acinar/tubular, microcystic/macrocystic, rarely pseudopapillary. Stroma: ussually nonspecific, without desmoplasia or inflamatory modifications; may be fibromyxoid, with calcification/ossification. **Hypervascular tumor**: ramified network of small calibre, thin-walled vessels. **High grade features**: rhabdoid/sarcomatoid differentiation.	**POSITIVE**: **PAX8** (nuclear, ~100%); **CAIX** (diffuse membranous/box-like, 75–100%); proximal tubular antigens (also seen in normal cells): **CD10** (membranous, 82–94%) and **RCCm** (cytoplasmic and membranous, 72–84%); epithelial markers (**AE1/AE3, CAM 5.2, EMA**); **Vimentin** (cytoplasmic and membranous). **NEGATIVE**: **CK7** (focally positive or patchy in high grade areas or cystic components); **CK20**; **AMACR**; **34βE12**; **CD117**; **HMB-45**; **TFE3/TFEB**; **Cathepsin-K**; **GATA3**; **MelanA**; **Inhibin**.	**SPORADIC**: 3p loss/deletions or biallelic alteration of VHL gene (3p25) by mutation or hypermethylation (80–98%). ccRCC tumor suppressor genes harbored by 3p locus: KD-M6A (or UTX), KDM5C (or JARID1C), SETD2 and PBRM1. Loss of chromosome 4p, 8p, 9p, 14q. Gain of chromosome 5q. Mutations in BAP1 and PBRM1 genes. **FAMILIAL**: Von Hippel-Lindau disease; constitutional chromosome 3 translocations.
**Papillary (p) RCC Type 1**	**Hypovascular tumor**. **Cellularity**: small cuboidal cells; uniform, diminished, pale/basophilic cytoplasm; hyperchromatic nuclei, absent nucleoli, lower grade than type 2; foamy macrophages and psammoma bodies are usually encountered. **Architecture**: single layer of of cells around fibrovascular cores (papillary), tubules and glomeruloid structures.	**POSITIVE**: **PAX8**; **CK7** (diffuse, but strong); **EMA** (polarized expression); **AMACR**; **AE1/AE3**; **CD10**. **NEGATIVE**: **CAIX**; **GATA3**; **p63**; **34βE12**; **TFE3/TFEB**; **Cathepsin-K**.	**SPORADIC**: Activating mutations or amplifications of MET proto-oncogene in >80% of sporadic cases. Gains in chromosomes 3, 7, and 17. **FAMILIAL**: Hereditary papillary renal carcinoma (HPRC).
**pRCC Type 2**	**Cellularity**: abundant eosinophilic cytoplasm; frequent nuclear atypia with clearly visible nucleoli (usually ISUP grade ≥3) and nuclear grooves; intracytoplasmic hemosiderin; possible areas of clear cytoplasm; less frequent foamy macrophages and psammoma bodies than type 1. **Architecture**: papillary, with pseudostratified layers of large cells; variable necrosis.	**POSITIVE**: **PAX8**; **CD10**; **AMACR**; **Topoisomerase II alpha.** **NEGATIVE**: **CAIX**; **CK7** (or patchy positive); **EMA** (or focal positive); **p63**; **HMB45**; **MelanA**; **Cathepsin-K**; **34βE12**; **GATA3**; **TFE3/TFEB**.	**SPORADIC**: Less consistent than type 1. Heterogeneous, losses or gains of chromosomes 1, 3, 4, 5, 6, 8, 9, 10, 11, 15, 18, and 22. NRF-ARE2 pathway amplification. 8q gains and allelic imbalance of 9q13 (prognostic significance). In advanced stages, CDKN2A/B (18%), TERT (18%), NF2 (13%) and FH (13%) are commonly altered. **FAMILIAL**: FH gene (1q42–43) mutation in HLRCC.
**Chromophobe (ch) RCC**	**Cellularity**: sharply defined, pale cells; “plant-like” cell borders (“vegetable cells”); wrinkled and angulated, irregular nuclei, with coarse chromatin (“raisinoid”); frequent bi/multinucleation and perinuclear halo (koilocytic atypia), with rare mitotic figures; Fuhrman/WHO nuclear grading has no prognostic value and is discouraged. 2 types of cellular morphologies: Type 1—large, polygonal cells, hard cell borders, abundant cytoplasm with reticular pattern (classical variant); Type 2—smaller cells with finely granular eosinophilic cytoplasm (predominant in eosinophilic variant). **Architecture**: confluent, solid growth with nests, sheets or alveoli/trabeculae; minimal stroma, composed of incomplete fibro-vascular septae around solid sheets; minimal vasculature.	**POSITIVE**: **PAX8**; **Hale colloidal iron** (histochemical stain; diffuse and strong, reticular); **CK7** (diffuse and strong); **CD117** (membranous); **Ksp-cadherin** (+/−); **GATA3**(+/−); **E-cadherin**; **claudin7** (distal nephron marker); **EMA** (diffuse cytoplasmic); **LMWCK (CK8/CK18)**; **RCCm**; **CD10**; **Parvalbumin** (calcium binding protein); **Cytochrome c oxidase**; **DOG1**; **Progesteron Receptor** (90% of cells); **PDL1 22C3** (in a minority of cases). **NEGATIVE**: **Vimentin** (or weak); **CAIX**; **AMACR**; **N-cadherin**; **Cathepsin-K**; **HMB-45**; low **Ki67** labeling index; **Cyclin D1**.	**SPORADIC**: Multiple losses of whole chromosomes: 1, 2, 6, 10, 13, 17, 21 or Y. DNA rearrangement breakpoints within the TERT promoter region. Mutated TP53 and PTEN in 10–30% of cases (TCGA cohort) and NRAS, mTOR and TSC1/TSC2 in ~5%. Mutations in mitochondrial DNA most commonly affect MT-ND5, with increased expression of genes encoding Krebs cycle enzymes. **FAMILIAL**: Birt-Hogg-Dube (BHD) syndrome—FLCN gene mutation (17p11.2)
**Clear Cell Papillary (ccp) RCC**	**Cellularity**: clear cytoplasm; characteristic linear arrangement of nuclei away from the basal aspect of cells; low nuclear grade (Fuhrman grade 1–2). **Architecture**: variable mixture of cystic, branched tubular, solid and papillary components; papillae often tightly packed into anastomosing clear cell ribbons or projecting into cystic spaces; fibrous capsule and variable amounts of hyalinized or sclerotic stroma that may separate the tumor into nodules; never encountered: foamy macrophages, vascular invasion, oxalate crystals, necrosis.	**POSITIVE**: **PAX8**; **CK7** (strong and diffuse); **CAIX** (diffuse membranous or characteristic “**cup shaped**” distribution—lack of staining along luminal aspect); **AE1/AE3**; **CAM 5.2**; **Vimentin**; **EMA**; **GATA3** (+/−); **34βE12**. **NEGATIVE**: **AMACR**; **CD10**; **RCCm**; **TFE3/TFEB**; low **PCNA**; **CD117**; **Cathepsin-K**; **HMB-45**.	**No specific genomic imbalances.** Lacks genetic modifications of classic pRCC (no +7/+17 or -Y) and/or classic ccRCC (no -3/-3p). Activation of hypoxia inducible factor (HIF) pathway, with underexpressed VHL transcripts, but without typical VHL mechanism (no VHL gene mutations/promoter hypermethylation). **May appear in VHL disease.**
**Collecting duct carcinoma (CDC)**	**Cellularity**: irregular channels, lined with high-grade cuboidal to hobnail cells, with eosinophilic cytoplasm; pleomorphic kariomegaly, with visible nucleoli and coarse chromatin; abundant mitosis. **Architecture**: the tumor is complex, infiltrative, and poorly circumscribed, composed of cords, tubules, tubulopapillary or tubulocystic structures, within an inflammatory-desmoplastic stroma; intracytoplasmic/intraluminal mucin, microcystic changes from dilation of the tubular structures and sarcomatoid transformation may be present. Tumor-adjacent tubular epithelium, lining collecting ducts, may appear dysplastic.	**POSITIVE**: **PAX8/PAX2**; HMWCK (**34βE12**); LMWCK (**CK7**, **CK8/18**, **CK19**); Ulex europaeus lectins (**Ulex-1**); peanut lectin agglutinin (**PNA**); **Mucin** (strong); **Vimentin**; **EMA**; **S100A1**; **INI-1/BAF47** retained. **NEGATIVE**: **p63**; **Uroplakin II**; **CD10**; **AMACR**; **E-cadherin; CAIX**; **OCT3/4**; **CD117**; **GATA3**.	Lacking loss of 3p or trisomies 7 and 17. HER2/neu amplifications in 45% of cases. Genomic alterations in NF2 (29%), SETD2 (24%), SMARCB1 (18%) and CDKN2A (12%). Recurrent somatic single nucleotide variants in ATM, CREBBP, PRDM1, CBFB, FBXW7, IKZF1, KDR, KRAS, NACA, NF2, NUP98, SS18, TP53 and ZNF521. SLC7A11 (cisplatin resistance associated gene), overexpressed in 80% of cases.
**Renal medullary carcinoma (RMC)**	**Cellularity**: cohesive groups of pleomorphic tumor cells, with vacuolated eosinophilic cytoplasm, that often displaces or indents the hyperchromatic, enlarged nuclei; nuclear membranes are often irregular, with coarse or vesicular chromatin and prominent nucleoli; rhabdoid traits are frequent; abundant intratumoral neutrophils and lymphocytes at tumor rim; abundant sickled erythrocytes (drepanocytes) may be pathognomonic. **Architecture**: multiple distinct morphologic patterns—reticular; microcystic and adenoid cystic-like; tubular, glandular, tubulopapillary and solid (overlapping with CDC patterns); hemorrhagic and geographic necrosis; angiolymphatic invasion, desmoplastic stroma and infiltrative borders.	**POSITIVE: CAM 5.2**; **AE1/AE3**; **CK7/CK20** (may be variable); **Vimentin**; **EMA** and **CEA**; **p53**; **PAX8**; **OCT3/4**; **Ulex-1** (focally positive in a minority of cases); vascular endothelial growth factor (**VEGF**) and hypoxia inducible factor (**HIF**) may be strongly positive. **NEGATIVE:** Loss of **INI-1/BAF47** expression; **colloidal iron**; **PAS**; **desmin**; **34βE12**; **GATA3**	Prevalent loss of SMARCB1/INI1, a major driving feature, identifiable by IHC. Hypoxia inducible factor and VHL abnormalities. ALK rearrangement with vinculin (VCN) fusion. DNA topoisomerase II amplification. Rarely, vinculin—ALK fusion (young patients, less aggressive). Associated with sickle cell trait (monosomy 11—beta globin gene is at end of 11p).
**Succinate dehydrogenase deficient renal (SDHD) RCC**	**Cellularity**: flocculent, pale, eosinophilic, cytoplasmic vacuolation, with a wispy/bubbly appearance and low grade nuclei, with smooth nuclear contours and fine chromatin, with no nucleoli, represents a characteristic finding and must be present at least focally. **Architecture**: well circumscribed tumor or with a “pushing” border, commonly entrapping tubules; solid, nested or tubular growth pattern with scattered cysts containing eosinophilic material; necrosis, sarcomatoid change and areas with higher grade nuclei may also be present; variant morphologies have been rarely reported. Shares features with chRCC, oncocytoma, ccRCC and pRCC type 2.	**POSITIVE**: **PAX8**; focal **pancytokeratin** and **CAM 5.2**; **EMA**. **NEGATIVE**: Loss of **SDHB** IHC staining (indicates disruption of the mitochondrial complex 2 for any reason, not just SDHB gene mutation and caution regarding overinterpretation of negativity in tumors with very clear cytoplasm is essential); **CK7**; **CK20**; **AE1/AE3**; **CAIX**; **RCCm**; **CD117** (mast cells only); **Vimentin**; **S100A1**; **TFE3/TFEB**; **neuroendocrine markers**; minimal **AMACR** staining.	Wild-type VHL, PIK3CA, AKT, mTOR, MET or TP53 genes. Genes for succinate dehydrogenase subunits (SDH-A, -B, -C, -D), encode protein components of mitochondrial complex II, linking the Krebs cycle with the electron transport chain, being involved in most cases of SDHD RCC. Germline mutations of SDH-A, SDH-B (1p36.13), SDH-C (1q23.3), SDH-D(11q23.1), SHDAF2, determining double hit inactivation, leads to dysfunction of mitochondrial complex II, increased oxidative stress, genomic injury and HIF1α stabilization.
**Microphtalmia family translocation (MiT) RCC—Xp11/t(6;11)**	**Cellularity**: voluminous cytoplasm and high-grade nuclei, with frequent psammoma bodies and occasional melanin pigment, similar to a pigmented perivascular epithelioid tumor—(PEC)coma. t(6:11) rearranged carcinomas are characteristically biphasic, with small cells clustered around basement membrane material (reminiscent of Call-Exner bodies in adult granulosa cell tumor) and larger epithelioid cells. **Architecture**: usually papillary and solid alveolar growth pattern, composed of clear to eosinophilic, discohesive pseudostratified cells.	**POSITIVE**: **TFE3/TFEB** (strong nuclear staining, but difficult to standardize on automated platforms; FISH assays are more reliable)—weak TFE3 staining in adults may not be specific; **PAX8**; **Cathepsin-K** (~50%, cytoplasmic); **CD10**; **AMACR**; **Vimentin**; **E-cadherin**; melanocytic markers (**HMB45** and **MelanA**), are common for **t(6:11)** carcinomas, but always focal, and infrequent for **Xp11**, which usually manifest variable **CD117**. **NEGATIVE**: Variable **cytokeratin** (only 30–50% positive, less than other RCC types) and **EMA** (50%, frequently only focal); **CAIX** usually negative except areas of necrosis; **CK7**; **34βE12**; **CD45**; **calretinin**; **smooth muscle actin**.	Fluorescence in situ hybridization (FISH) with a TFE3/TFEB breakapart probe is highly sensitive and specific, offering the final diagnosis when morphology and IHC are inconclusive. The TFE3 gene (on Xp11) has been reported to have multiple translocation gene partners, most commonly, ASPL (17q25) and PRCC (1q21), and less commonly NONO (Xq12), PSF/SFPQ (1p34), CLTC (17q23). t(6;11)(p21;q12), a translocation between TFEB and MALAT1 genes, results in overexpression of TFEB. t(X;17)(p11.2;q25), with balanced translocation of TFE3 gene at Xp11.2 and ASPL gene at 17q25, is present in renal neoplasms, whereas in alveolar soft part sarcoma, this translocation is unbalanced, der(17)t(X;17)(p11.2;q25). Melanotic Xp11 RCC and PSF/SFPQ-TFE3 (PEComa), may share the same genetic abnormalities.
**Acquired cystic disease-associated (ACDA) RCC**	**Cellularity**: moderately cellular, papillary clusters of polygonal to columnar cells with abundant eosinophilic granular cytoplasm, round and central nuclei, finely granular chromatin, prominent, central, grade 3 nucleoli; sometimes with prominent clear cell cytology. **Architecture**: cribriform, microcystic or sieve-like layout; intratumoral calcium oxalate crystals are very common, but not mandatory for diagnosis; nodules arising from cyst walls or masses separated from cysts may be encountered.	**POSITIVE: No specific IHC profile** is required for diagnosis. **CD10**; **AE1/AE3**; **AMACR**; **NEGATIVE: EMA**; **CK7** (but may be focally positive).	Comparative genomic microarray and FISH studies reveal gains and losses of multiple chromosomes. Gains of sex chromosomes and gains of 3, 7, 16, 17, with a high prevalence of gains of Y, 3 and 16, distinguishing ACDA RCC from pRCC, which also has gains in chromosomes 7 and 17. VHL gene alterations. Chromosome 3p deletion.
**Multilocular cystic clear cell renal neoplasm of low malignant potential (MCLMP) RCC**	**Cellularity**: single layer of clear cells lining thin fibrous septae or in small clusters; low grade nuclei without nucleoli (ISUP grade 1–2); bland clear cells in septa may be mistaken for lymphocytes (vascularity is important). **Architecture**: cyst lining may be denuded and, in rare cases, cyst lining may be multilayered, with granular cytoplasm cells and small intracystic papillations; septa may contain calcification or ossification; no expansile growth of clear tumor cells/solid nodules; no necrosis, vascular invasion or sarcomatoid change.	**POSITIVE**: **PAX8/PAX2**; **CA IX**; **EMA**; variable **CK7**. **NEGATIVE**: **AMACR** (negative in 80% of cases).	Genetically related to ccRCC, with 74% of cases demonstrating 3p loss and VHL mutations identified in 25%.
**Tubulocystic** **(TC) RCC**	**Cellularity**: tubules/cysts are lined by a single layer of flattened, cuboidal or columnar cells; hobnailing may be present, with modest to abundant amounts of eosinophilic cytoplasm, resembling oncocytoma cell; uniform, round nuclei, with distinct nucleoli (ISUP grade 3); minimal mitotic activity and atypia; very rare necrosis. **Architecture**: mixture of closely packed tubules and variably sized cysts, with overall low-grade morphology; cysts are separated by fibrous septa; no desmoplasia or cellular stroma; frequently associated with papillary cell neoplasms.	**POSITIVE**: **CK7**; **AMACR**; **Vimentin**; **EMA**; **PAX8**; fumarate hydratase (**FH)**; **Mucin**; keratins (**AE1/AE3**, **Cam 5.2**, **CK8/18**, **CK19**); variable **34βE12**; **CD10**. **NEGATIVE**: **2 succino-cysteine**, **CA IX** and **CD117**.	Distinct molecular signature from other RCCs. Frequently, gains of chromosomes 7 and 17, and loss of Y, similar to pRCC. Mutations in 14 different genes have been documented by targeted, next generation sequencing, most frequently (60% of cases) in ABL1 and PDFGRA genes.
**Mucinous tubular and spindle cell (MTSC) RCC**	**Cellularity**: relatively uniform, bland, low-grade cuboidal cells, with round to oval nuclei and focally vacuolated, eosinophilic cytoplasm, within strands of metachromatic stromal tissue, which transition into anastomosing spindle cells; may present clear cells and focal clusters of foamy macrophages; rare high-grade nuclei may be present. **Architecture**: well circumscribed epithelial tumor, partially surrounded by a rim of compressed fibrous tissue; long tubular/cord-like growth pattern; myxoid and bubbly stroma, with abundant extracellular mucin, highlighted by Alcian blue (although some cases may be mucin poor); may manifest well-formed papillae, necrosis, rarely neuroendocrine differentiation or sarcomatoid change; usually, infiltrative growth, desmoplasia, inflammation, hobnail epithelium, and/or cysts are not encountered.	**POSTIVE: PAX2/PAX8**; **EMA** (95%); **AMACR** (93%); **AE1/AE3**; **E-cadherin**; **LMWCK**: **CK7** (81%); **CK 8/18**; **CK19**; Neuron Specific Enolase (**NSE**) and either **Chromogranin** or **Synaptophysin.** **Histochemical stains:** Periodic Acid-Schiff **(PAS)** (basal lamina around tubules), **Alcian blue** (mucin). Occasionally, HMWCK: **34βE12** (15%); **vimentin**; **Ulex** or **CD10** (15%). **NEGATIVE: CA IX** (positive next to necrosis or focal cytoplasmatic in high-grade areas); **GATA3**; **p63**; **RCCm** (positive in 7%); **Villin**; **Ki67** (<1%).	Seemingly lacking the characteristic genetic modifications of classic pRCC (trisomies of chromosomes 7/17 or loss of chromosomes Y). Usually hypodiploid, with multiple chromosomal losses (-1, -4, -6, -8, -9, -13, -14, -15, -22), even hypertriploid in some cases, but with no identifiable pattern.
**Hybrid oncocytic-chromophobe (HOCh) RCC**	**Cellularity**: usually, dual population of eosinophilic cells—**oncocytic** (medium sized, round cell, with granular eosinophilic cytoplasm and concentric round nucleus, low nuclear:cytoplasmic ratios, prominent nucleolus) and **chromophobe** (large, polygonal, “plant-like” cell, with a distinct cell membrane, containing flaky eosinophilic cytoplasm, often with a perinuclear halo and an irregular “raisinoid” wrinkled nucleus), with a third cell subtype, manifesting overlapping cytonuclear features of both oncocytic and chromophobe morphology, being sometimes encountered; mitotic rate is very low. **Architecture**: well circumscribed, non-infiltrative, intrarenal tumor, with a solid alveolar and cystic architecture; may manifest vascular invasion and, rarely, necrosis.	**POSITIVE**: **CK7** (may be focal); **AE1/AE3**; **Parvalbumin**; **Antimitochondrial Antigen**; **EMA**; **E-cadherin** (most); **CD117**; **S100A1**; **CD82**; **Vimentin** (few cases); **Hale colloidal iron** (stains apical/luminal oncocytic cells and, often, intracytoplasmic in chromophobe cells. **NEGATIVE**: **AMACR**; **CK20**; **CD10** and **CA IX**.	**SPORADIC**: May present numerous molecular anomalies (both mono- and polysomies) of chromosomes 1, 2, 6, 9, 10, 13, 17, 21, and 22. Lack of mutations in the VHL, c-kit, PDGFRA, and FLCN genes. **FAMILIAL**: May be associated with BHD, autosomal dominant syndrome, characterized by a genetic abnormality on chromosome 17p11.2, leading to a mutation in the FLCN gene.

**Table 2:** Differential diagnosis-driven IHC panels and profiles for morphological RCC traits [6,64].

Predominant Morphological Trait	Specific Panel	Entities Entering Differential Diagnosis with Corresponding IHC Profiles
**Clear cell** **Population**	**CAIX** **CK7** **CD117** **Cathepsin-K** **HMB-45**	**ccRCC**: CAIX(+, diffuse membranous), CK7(−), CD117(−), Cathepsin-K(−), HMB-45(−) **ccPRCC**: CAIX(+, cup-like pattern), CK7(+), CD117(−), Cathepsin-K(−), HMB-45(−) **classic chRCC**: CAIX(−), CK7(+, cytoplasmic), CD117(+, membranous), Cathepsin-K(−), HMB-45(−) **eAML**: CAIX(−), CK7(−), CD117(−), Cathepsin-K(+, cytoplasmic), HMB-45(+, cytoplasmic) **MiT-TFE RCC**: - **Xp11/TFE3**: CAIX(+/−, focal), CK7(−), CD117(+/−), Cathepsin-K (+, 50%, cytoplasmic), HMB-45(−) - **t[6;11]/TFEB**: CAIX(+/−, focal), CK7(−), CD117(−), Cathepsin-K(+, cytoplasmic), HMB-45(+, focal)
**Papillary** **Component**	**CAIX** **CK7** **AMACR** **Cathepsin-K** **34βE12** **TFE3/TFEB**	**ccRCC with papillary growth**: CAIX(+, membranous), CK7(−), AMACR(−), Cathepsin-K(−), 34βE12(−), TFE3/TFEB(−) **pRCC “type I”**: CAIX(−), CK7(+), AMACR(+), Cathepsin-K(−), 34βE12(−), TFE3/TFEB(−) **pRCC “type II”**: CAIX(−), CK7(+/variable), AMACR(+), Cathepsin-K(−), 34βE12(−), TFE3/TFEB(−) **ccPRCC**: CAIX(+, cup-like pattern), CK7(+, diffuse), AMACR(−), Cathepsin-K(−), 34βE12(−), TFE3/TFEB(−) **MiT-TFE RCC**: CAIX(variable, focal), CK7(−), AMACR(+), Cathepsin-K(+, 50%), 34βE12(−), TFE3/TFEB(+, but difficult to standardize on automated platforms, requires FISH assays)
**Solid growth pattern**	**CK7** **AMACR** **WT-1** **CD57**	**Solid pRCC “type I”**: CK7(+), AMACR(+), WT-1(−), CD57(−) **Metanephric adenoma**: CK7(−)/isolated cells, AMACR(−), WT-1(+, nuclear), CD57(+) **Wilms’ Tumor**: CK7(−)/isolated cells, AMACR(−), WT-1(+, nuclear), CD57(−)
**Cytoplasmic** **Eosinophilia**	**CD117** **CK7** **Ksp-cadherin** **HMB-45** **Cathepsin-K**	**Oncocytoma**: CD117(+, membranous), CK7(−), Ksp-cadherin(+), HMB-45(−), Cathepsin-K(−) **Eosinophilic chRCC**: CD117(+, membranous), CK7(+, but variable), Ksp-cadherin (+, mostly), HMB-45(−), Cathepsin-K(−) **Oncocytic PRCC**: CD117(−), CK7(+, focal), Ksp-cadherin (unknown), HMB-45(−), Cathepsin-K (unknown) **Oncocytic AML**: CD117(−), CK7(−), Ksp-cadherin(−), HMB-45(+, focal), Cathepsin-K(−)
**Sarcomatoid** **growth pattern**	**Vimentin** **CAIX** **PAX8** **CK7** **34βE12** **GATA3** **p63**	**ccRCC**: Vimentin(+), CAIX(+) membranous, PAX8(+), CK7(−), 34βE12(−), GATA3(−), p63(−) **pRCC**: Vimentin(+), CAIX(−), PAX8(+), CK7(focal/-), 34βE12(−), GATA3(−), p63(−) **chRCC**: Vimentin(+), CAIX(−), PAX8(+), CK7(+), 34βE12(−), GATA3(−), p63(−) **MTSC**: Vimentin(+), CAIX(−), PAX8(+), CK7(+), 34βE12(variable), GATA3(−), p63(−) **Urothelial**: Vimentin(+), CAIX variable/mostly(−), PAX8 mostly(−), CK7(+), 34βE12(+), GATA3(+), p63(+) **Sarcoma**: Vimentin(+), CAIX(−), PAX8(−), CK7(−), 34βE12(−), GATA3(−), p63(−)
**Distal nephron origin**	**INI-1/BAF47** **OCT4** **GATA3** **PAX8**	**CDC**: INI-1/BAF47 (+, and—in 15%), OCT4(−), GATA3(−), PAX8(+) **RMC**: INI-1/BAF47 (−), OCT4(+), GATA3(−), PAX8(+) **Urothelial**: INI-1/BAF47 (+), OCT4(−), GATA3(+), PAX8(−, but + in 20%)

Novel PAX8 antibodies (PAX8R1), to address the specificity issues of polyclonal PAX8 preparations, have been developed. They bind to the C-terminal of PAX8, targeting amino acids 318–426, which are highly divergent among PAX proteins, thus abolishing cross-reactivity with other PAX species [162].

Further nuancing of IHC differential diagnosis in mRCC involves markers almost always negative in RCC, such as pulmonary marker TTF-1, the intestinal marker homeobox protein CDX2, p63, prostate-specific antigen, and estrogen receptor, which will be useful in excluding other carcinomas that may manifest cross-positivity for PAX8. Positive staining with any of the aforementioned markers represents a strong argument against the diagnosis of mRCC [6]. Another useful distinction, between urothelial and renal epithelial origins, can be made using GATA3, a transcription factor involved in cell differentiation and proliferation in a variety of tissues and cell types, which will be expressed in most urothelial tumors, but not in RCCs [163,164].

Other supportive IHC markers of mRCC, currently in common practice—cluster of differentiation (CD)10, RCC marker antigen (RCCm), Kidney-Specific Cadherin (Ksp-cadherin)—manifest inadequate specificity and are not usually indicated or useful, outside of very specific, punctual, diagnostic subtleties.

RCCm stains a proximal tubular antigen and demonstrates focal labeling in approximately 80% of RCC [165,166], yet with notoriously poor specificity, seeing as it also labels many other carcinomas (breast, lung, colon, and of adrenal origin). It is useful in differentiating clear cell RCC (ccRCC) from ovarian clear cell carcinoma, as PAX8 would be positive in both tumors, whereas RCCm would be positive only in the renal neoplasm. Moreover, PAX8 is negative in adrenal cortical neoplasms, which preferentially stain for steroid factor-1 [167].

Another proximal tubular marker, CD10, also manifests high sensitivity, but again very low specificity for RCC, as lung, bladder, colon, and ovarian carcinomas all label for CD10 [168]. However, CD10 fairly consistently labels ccRCC, thus CD10 negative metastatic lesions represent an argument against this diagnosis for the primary tumor.

Lastly, Ksp-cadherin, a distal tubular marker, manifests high sensitivity for chromophobe RCC (chRCC), although it is not so useful in the metastatic diagnosis context, as the chromophobe variant rarely disseminates systemically [169]. Even so, at least focal staining can also be seen in other renal tumor variants, including high-grade ccRCC.

## 5. RCC Molecular Pathology and Clinical Applications for Emerging RCC Biomarkers

In spite of the substantial improvements achieved so far, regarding RCC detection, diagnosis (comprehensive clinical definitions, pathological molecular characterization, nuanced differential diagnosis and subtyping), and systemic treatment modalities, systemically disseminated RCC remains an incurable disease, while RCC mortality rates continue to rise. Thus, further investigations into RCC carcinogenesis are urgently needed to better comprehend the intimate mechanisms involved in disease occurrence and progression, and to possibly identify adequate biomarkers for RCC screening and risk stratification [170].

### 5.1. RCC Carcinogenesis, Disease Progression, and Prognosis Assessment

For decades now, the scientific community has struggled to find predictive tools for the adequate and individualized characterization of RCC prognosis. Despite significant progress in RCC molecular pathology, RCC prognosis is still best evaluated by using traditional parameters, evaluating tumor anatomy and loco-regional extension/systemic dissemination (TNM classification/individual components), tumor histology (nuclear grade, specific subtype, necrosis, lympho-vascular, and collecting system invasion [171]), and clinical status (performance scores, local symptomatology, cachexia, anemia, platelet/neutrophil/lymphocyte count, C-reactive protein, and albumin levels [172,173,174,175,176,177,178]). Even though multiple biomarkers have been proposed and investigated [179,180,181,182], due to the retrospective nature of these inquiries, the small size of cohorts evaluated, limited availability of clinical information, and the lack of additional investigations for validation of results reported, no single biomarker (or panel of biomarkers) has yet emerged as reproducible and useful in the clinical setting for predicting RCC progression and/or response to systemic treatment [6]. Regardless, as was the case for breast and lung carcinomas [183], melanoma, and hematopoietic neoplasms, the recent genomic and molecular insights into signaling pathways involved in RCC metabolism and carcinogenesis, particularly for ccRCCs [81,184], will be followed by molecular pathway-targeted therapeutic interventions and clinical trials, which, in turn, will yield novel integrative RCC management tools, able to better predict RCC outcomes, nuance systemic treatment options, and facilitate more objective, treatment response prediction based, therapeutic strategy elaboration and personalization [185,186,187,188,189].

As we enter a new era of personalized medicinal oncology, long-term, large-scale, and carefully coordinated genomic sequencing investigations, i.e., The Cancer Genome Atlas (TCGA), have already achieved full genome mapping for most cancers. Thus far, total RCC genome analysis has been achieved [190], resulting in the identification of highly specific RCC signature mutational patterns, mainly involving the *von Hippel*–*Lindau* (*VHL*) and *Polybromo 1* (*PBRM1*) genes [191]. Additionally, each individual conventional RCC subtype (ccRCC, pRCC, and chRCC) has shown specific significantly mutated gene clusters comprised of RCC signature gene mutations (*VHL*/*PBRM1*), together with other recurrent mutations in non-specific genes, i.e., *SETD2*, *KDM5C*, *PTEN*, *BAP1*, *MTOR*, and *TP53* [81], which apparently hold some predictive value regarding RCC prognosis. Comparative analysis of these significantly mutated gene clusters within conventional RCC subtypes showed that only *TP53* and *PTEN* (*phosphatase and tensin homolog*) mutations were encountered ubiquitously, in all conventional RCCs evaluated (ccRCC/pRCC/chRCC), yet prognostic value remained RCC subtype-specific. Thus, *TP53* mutations in ccRCCs and pRCCs were indicative of diminished survival rates, while *PTEN* mutations associated the same decrease in survival, but only for chRCCs [190].

Furthermore, it seems that, through various mechanisms, i.e., 3p chromosomal loss, gene mutations, or epigenetic alterations, the VHL metabolic pathway usually becomes inactivated in virtually all ccRCCs. Consequentially, the hypoxia-inducible factor (HIF) pathway undergoes uninhibited hyper-activation, upregulating downstream molecular mediators, such as carbonic anhydrase IX(CAIX), and vascular endothelial growth factor (VEGF) [81,181,184], which will then proceed to drive carcinogenesis, stimulating tumor growth and progression. Additionally, as reported within TCGA, ~30% of ccRCCs demonstrate altered signaling within phosphoinositide 3-kinase (PI3K) pathways, of which ~6% specifically show mutation-driven activation of the mammalian target of rapamycin (*MTOR*) gene. Unsurprisingly, targeting these metabolic pathways in advanced ccRCCs using VEGF/mTOR inhibitors has demonstrated therapeutic value, improving disease-free survival rates, albeit with no effect on overall survival [185,186,189]. Even so, for the time being, no reliable IHC biomarkers have been identified amongst molecules within the VHL/mTOR pathways capable of adequately predicting response to targeted systemic therapy. As the sole exception worth mentioning, CAIX, known to be inherently expressed in ccRCCs [192,193,194,195,196,197], has shown some predictive value, as lower expression rates are associated with unfavorable outcomes, whereas high density CAIX expression, i.e., in >85% of tumor cells, may be indicative of therapeutic response to combined therapy (interleukin-2 + mTOR inhibitors), but these results still lack validation studies and remain debatable [6].

Moreover, regarding ccRCCs exclusively, a distinct set of recurring mutations at the level of multiple chromatin remodeling and histone modifying genes has been reported. Interestingly, these genes are located on chromosome 3p, in the proximity of the pathognomonic VHL sequence [192,198,199,200,201,202] (see Table 1). Among these genes, *PBRM1* is mutated in a third of ccRCCs, according to TCGA data, being the second most common gene affected by (cc)RCC signature mutations, after *VHL* (~52%) [191]. Although incapable of predicting overall survival and/or poor outcomes, *PBRM1* mutations are seemingly indicative of advanced local extension (extrarenal/pT3a), in otherwise inconspicuous small renal masses, predicting perinephric/sinus fat/small vessel RCC invasion, which was not apparent on contrast enhanced imaging [6]. Conversely, mutations of other neighboring 3p genes, namely *Histone-Lysine N-Methyltransferase* (*SETD2*) and *Ubiquitin Carboxyl-Terminal Hydrolase* (*BAP1*), have shown significant correlations with higher Fuhrman grades and unfavorable clinical outcomes [192,201,202]. Comparatively, it seems that *BAP1*-mutant RCCs have more unfavorable clinical outcomes than *PBRM1*-mutant RCCs [192].

Beyond the 3p chromosomal loss, mainly characteristic of ccRCCs, loss of 9p and 14q have also consistently associated with more aggressive RCC behavior and poor survival rates [92,203,204]. A recently proposed genetic classification of RCCs and matching metastases incriminates chromosomal complexity as the main predictor for systemic dissemination, RCC aggressiveness and overall survival [203], indicating that a massive chromosomal level genomic injury, concomitantly interfering with the expression patterns of hundreds of individual genes, may represent the supportive molecular substrate for the elaborate metastatic-driving cascade of alterations incurred during RCC progression, impacting various functional phenotypes [205] and potentially facilitating RCC immune evasion [206]. In line with this complexity, a multifactorial 16 gene RCC signature cluster was defined for non-metastatic RCCs, which, albeit lacking external validation, showed disease-free survival predictive value [207].

In fact, ongoing integrative molecular profiling efforts, focused on ccRCC pathophysiology, have highlighted the fact that ccRCC is much more than just the aberrant proliferation of renal cellularity, but rather a fundamentally metabolic disease, defined by specific key genetic mutations in target metabolic pathways, resulting in ccRCC metabolic reprogramming, throughout various cellular processes—cellular oxygenation (*VHL*), epigenetic modifications (*PBRM1*, *SETD2*, *BAP1*), and growth factor mediated intracellular signaling (*MTOR*). These major ccRCC metabolic alterations are evolutionarily advantageous for tumor cells, mainly translating to increased glycolysis, enhanced pentose phosphate pathways, downregulation of the tricarboxylic acid cycle, and augmented glutamine uptake. Thus, metabolic reprogramming allows ccRCC cells to survive in a hostile microenvironment, despite energy/nutrient deprivation and hypoxia, and to synthesize new building blocks for proliferation (DNA strands, proteins, lipoproteic cellular membranes), while bypassing host immunosurveillance and counteracting oxidative stress [208].

Hereditary germ-line mutations in proximal tubular cells, but also acquired somatic mutations within ccRCC’s natural evolution, represent the driving genomic forces behind the adaptation of ccRCC metabolism, important in both carcinogenesis, as well as disease progression. Thus, implicitly, the specific type of metabolic reprogramming which occurs is highly dependent on the ccRCC grade [209]. Specifically, aerobic glycolysis reprogramming will play a pivotal role in the initial stages of renal cancerogenesis, ensuring ccRCC tumor cell survival and proliferation via increased lactate production and tricarboxylic acid cycle downregulation [210]. Conversely, in later stages, alterations regarding the tryphtophan, glutamine, and fatty acid pathways will be heavily involved in allowing for tumor cell evasion of host immunosurveillance, as well as antioxidant responses and energy storage [208].

From a clinical perspective, the in-depth analysis of the biological implications of these aforementioned ccRCC metabolic alterations, alongside the quantification of the resulting modifications in expression levels of biochemical enzymes, substrates, intermediates and final metabolic products, derived from ccRCC metabolism reprogramming, may constitute diagnostic and prognostic biomarkers, as well as novel therapeutic targets. Thus, emerging proton nuclear magnetic resonance spectroscopy-based metabolomics have revealed, albeit within a limited ccRCC cohort, pathognomonic urinary metabolic profiles for ccRCC patients (increased levels of creatine, alanine, lactate, and pyruvate, with decreased levels of hippurate, citrate, and betaine), which distinguished them from healthy individuals and post-nephrectomy patients. Upon further analysis, most of these metabolites were linked to pathogenic processes—i.e., glomerular injury, renal inflammation, and renal necrosis/cell death. When comparing the urinary metabolome of the same ccRCC patients, prior to and post-nephrectomy, these pathogenic processes had apparently become inactive as a consequence of radical treatment [211].

Additionally, regarding the adaptive alterations of the energetic metabolism in ccRCCs, a negative and proportional correlation has been established between the expression levels of glycolytic enzymes and progression-free/cancer-specific survival rates, i.e., higher expression levels associate poor disease outcomes. Apparently, the oncogenic signaling pathways responsible for rerouting the glucidic metabolism in ccRCCs are also central promoters of ccRCC carcinogenesis and progression. More specifically, the enhanced flux of glucides metabolized through the pentose phosphate pathway, in association with the upregulation of glucose-6-phosphate dehydrogenase, promote both anabolic reactions and redox homeostasis in ccRCCs. Promisingly, an inhibitory intervention within this metabolic adaptation may serve as a novel ccRCC therapeutic target [212].

Focused and ongoing efforts to better understand ccRCC metabolic signatures, characterized by an anaerobic switch within the physiological energetic/glucidic cellular metabolism, towards the pentose phosphate pathway, have already proven to be fruitful, yielding multiple and seemingly promising novel RCC-associated molecular targets. Specifically, NADH dehydrogenase (ubiquinone) 1 alpha subcomplex 4-like 2 (NDUFA4L2), a HIF-1 target gene, encoding for a protein that attenuates mitochondrial oxygen consumption through inhibiting the electron transport chain Complex I, has been recently reported as being one of the most highly expressed genes in ccRCCs. NDUFA4L2 seemingly plays an important role within the process of ccRCC bioenergetic metabolic reprogramming (i.e., preferential activation of the pentose phosphate pathway, and impairment of mitochondrial activity), while also being involved in various other fundamental cellular processes (proliferation, migration and angiogenesis). Also noteworthy is the fact that NDUFA4L2 over-expression apparently stimulates ccRCC drug resistance, seeing as NDUFA4L2 knockdown results in decreased ccRCC cell viability, and improved cisplatin susceptibility, implying that this protein can regulate chemotherapy resistance in ccRCCs [213].

Conversely, despite both the well-established relationship between RCC stage at initial diagnosis and subsequent clinical aggressivity/overall survival (i.e., advanced stages associate poor outcomes and vice versa), but also the grade-dependent nature of RCC metabolic reprogramming patterns (i.e., alterations in glucidic [212] and/or lipidic [214] cellular processing), to date, the elaboration of grade-specific therapeutic modalities, targeting these metabolic particularities, has yet to be achieved. In an effort to address this lingering issue, a recent in vitro investigation, using low- and high-grade ccRCC derived cell cultures, analyzed the comparative molecular and cellular effects of selective inhibition of either glycolysis (with 2-deoxy-D-glucose/2-DG), or fatty acid oxidation (with Etomoxir), respectively. Grade-dependent modulation of lipid/glycogen storage was reconfirmed. Furthermore, the prevalence of aerobic glycolysis (Warburg effect), as the main source of cellular energy production, appears to be similarly dependent on ccRCC cellular differentiation. Interestingly, 2-DG exposure impaired cellular proliferation and viability in low-grade ccRCC and normal cortex cultures, whereas Etomoxir showed a cytostatic and cytotoxic effect only in high-grade ccRCCs. Clearly, the idea of a grade-dependent, metabolism-targeted, therapeutic strategy for ccRCC must be further investigated [209].

All in all, even though multiple retrospective and large molecular screening investigations have reported specific gene mutations and/or chromosomal alterations for ccRCC, which hold particular clinical implications, unfortunately, recently defined RCC subtypes are very scarcely investigated. Outside of conventional RCC subtypes, there is little to no information available regarding recurrent genomic lesions and their prognostic significance. Even among the plethora of investigated molecular markers, specific to ccRCC biology (CAIX, VEGF, HIF), proliferation/cell cycle (Ki67, p53, p21 [215], PTEN [216]), cellular adhesion (E-cadherin, CD44 [217,218]), immune response (osteopontin [219], CXCR4 [220], PD-L1 [221]), and epigenetic modifications (miRNA, gene methylations), none could produce more than level III evidence [222]. Indeed, for the most part, the aforementioned markers show prognostic associations and may have some additional, albeit still poorly defined, added value over current standard prognostic models, yet there are very little data to support the individual reports and offer external validation.

Ideally, beyond prognosis evaluation and systemic treatment case selection, after initial differential diagnosis, the ultimate oncological biomarker must also have cancer screening abilities, predicting the risk of disease occurrence even before carcinogenesis initiates. Promisingly, Kidney-Injury Molecule-1 (KIM-1), a glycoprotein specific to proximal tubular structures and well-established as a reliable urinary/plasmatic immunocytochemistry marker for the diagnosis of acute tubular necrosis/injury in acute renal insufficiency, has shown the ability to predict RCC occurrence, as early as five years before initial diagnosis [223,224]. Additionally, RCCs expressing higher levels of KIM-1 showed reduced patient survival rates [223]. Noteworthy is the fact that KIM-1, constitutively expressed in proximal tubular cellularity, can also be used as a IHC tool for RCC subtyping, indicating, when expressed, the aforementioned proximal tubule origin of the evaluated specimen, meaning ccRCC or pRCC [224]. To date, KIM-1 is seemingly the most multifaceted and nuanced RCC biomarker identified, with cheaper and more facile clinical applicability than previously described genetic markers, but similarly lacking external validation.

### 5.2. RCC Treatment Response Prediction

Essentially, all types of malignancies, regardless of carcinogenic origin, occur when a cell/population of cells, regardless of specific causal molecular pathology mechanisms, acquire the ability to evade immune cytotoxic control and divide uncontrollably. Therefore, implicitly, a certain degree of local immune response dysfunction within the tumor microenvironment (TME) is a prerequisite for the occurrence of any malignant tumor, i.e., a disparity between cytotoxic tumor-targeted responses versus tumor-promoting inflammation. In the case of RCCs in particular, an intensely neoangiogenic and immunogenic subset of epithelial tumors, TMEs are generally volatile and remarkably dynamic, manifesting extreme cellular pleomorphism. The main bioactive cellular populations consistently expressed in the RCC TME are as follows: (myo)fibroblasts, adipocytes, sporadic neuro-endocrine cells, immune and inflammatory response cells, and endothelial cells. This cellularity can be found dispersed within an equally bioactive extracellular supportive matrix. Thus, RCC cells must constantly interact with this amalgamation of cells with distinct origins (stromal and immune response) and metabolic profiles, either directly, via secretion of autocrine/paracrine-acting mediators, but also indirectly, during RCC proliferation, via the occurrence of hypoxic/necrotic events. This elaborate and extremely disruptive interplay ceaselessly reshapes and metabolically redefines RCC biology. The resulting heterogeneity will most likely prove to be essential for obtaining an accurate and complete understanding of RCC carcinogenesis, not to mention an integrative perspective upon the plethora of already described molecular mechanisms allowing for RCC progression and therapy resistance [170].

To date, the most significant breakthroughs achieved in RCC systemic therapy are a direct result of extensive assessment efforts of the biological significance of specific key inflammatory pathways within RCC metabolomics and in relationship to the RCC TME. As a result, multiple clinically relevant RCC-associated-antigens were identified and targeted therapeutic agents were subsequently developed (i.e., tyrosine kinase inhibitors and mTOR inhibitors). Subsequently, the RCC TME proved essential once again, within the process of defining and objectively quantifying the extent of RCC treatment responses. The meticulous evaluation of RCC TME treatment-induced modifications and the quantification of specific cellular TME constituents and the variations within their expression patterns after systemic treatment, facilitated the development of a more profound and nuanced understanding of these recently developed targeted systemic therapies, revealing their biological effects and specific molecular dynamics [225,226].

The ongoing effort to define the relationship more comprehensively between RCC cells and the various immuno-inflammatory host responses, has recently been additionally bolstered by an extensive analysis of the potential roles of complement system activation within the ccRCC TME, centered around the quantification of expression levels for Pentraxin-3 (PTX3), an innate immune regulator, both in ccRCC tumor samples and patient serum. PTX3 is able to activate the classical pathway of the complement system (C1q) and to release pro-angiogenic factors (C3a, C5a), thus stimulating ccRCC proliferation and dissemination. Furthermore, ccRCC patients consistently showed higher PTX3 serum levels, when compared to non-neoplastic controls. Additionally, higher PTX3 serum levels were strongly associated with higher Fuhrman grades, lymph node involvement, visceral metastases, and significantly diminished survival rates [227].

Conversely, RCC TME investigations focused on local immunosuppressive molecular mechanisms, responsible for RCC carcinogenesis and progression due to the facilitation of host cytoxic immune response evasion, have provided promising, albeit still unvalidated, predictive tools for the assessment of RCC prognosis and risk stratification. A recent investigation quantifying the stratified expression levels for individual constituents of the kynurenine (KYN) pathway, through which tryptophane is metabolized in ccRCCs reported the involvement of KYN pathway enzymes/catabolites in ccRCC carcinogenesis, defining both immune and non-immune mechanisms. The most useful parameter evaluated was the KYN-to-tryptophan ratio (KTR), which was able to accurately predict ccRCC aggressiveness, while also demonstrating prognostic significance regarding cancer-specific survival and progression-free survival [228].

Sustained RCC sequencing initiatives and big data processing are paramount for further pathway-targeted treatment development and a deeper understanding of RCC carcinogenesis. Furthermore, molecular characterization of anti-tumor immune responses vs. tumor progression facilitating inflammation will most likely offer a definitive cure for RCCs and, actually, for cancer in general. In fact, immunotherapy, the most recent oncotherapy, based on the novel principle of innate anti-tumor immune response activation, is the only available therapeutic modality which has the ability to target and destroy all types of tumor cells, regardless of cellular maturation, cell cycle, and metabolic status. More specifically, mitotically inactive, metabolically dormant, immature stem-like tumor cells are implicitly immune to other specific metabolic pathway-targeted systemic therapies, as they require tumor cells to manifest the targeted metabolic activity.

Importantly, in the first clinical trial of its kind, KEYNOTE-564 evaluated the benefits of adjuvant immunotherapy (Pembrolizumab) in RCC patients, post radical/partial nephrectomy, and reported a significant improvement in disease-free survival, as compared to placebo, among RCC patients with a high risk of disease recurrence [229]. Currently, multiple immune checkpoint inhibitors (ICIs), i.e., anti-PD1/PD-L1 and anti-CTLA4, have already been developed and subsequently evaluated in the context of advanced RCC and/or recurrence/treatment failure/salvage therapy, in large scale, comparative, and mostly still ongoing clinical trials. Currently, due to noteworthy improvements in survival rates for systemic disease, immunotherapy has been included in RCC management strategies, with various ICIs already being used almost routinely in clinical practice to treat advanced/recurrent RCCs. Even so, for the time being, the role of immunotherapy in RCC management is still being defined and nuanced, especially for the neoadjuvant and first line adjuvant setting. Consequentially, ethical issues have emerged, regarding risk of RCC progression under ICIs for localized RCCs and the still uncertain superiority over standard tyrosine kinase inhibitors (TKIs) in the first line for metastatic RCCs. To address these concerns, while also curtailing the still exorbitant treatment associated costs, a very meticulous and well-rounded case selection protocol must be developed and validated for RCC immunotherapy and/or for each specific ICI, ideally based on an objective treatment-response assessment, using tumor tissue-derived and/or systemic humoral immune-response predictive biomarkers.

Despite this burning need, to date, for metastatic RCC systemic therapy case selection, no specific molecular biomarker has shown treatment response predictive value [221,230,231] and, therefore, their routine use in clinical practice and therapeutic decision-making is discouraged. Even so, several predictive biomarkers have been investigated for metastatic RCC immunotherapy response assessment, yet only for individual ICIs and/or in very limited therapeutic associations and clinical contexts.

The JAVELIN trial (NCT02684006) identified specific gene signatures (immunomodulatory-associated vs. angiogenesis-associated gene clusters), which seemingly had the ability to accurately and differentially predict progression free survival (PFS) rates for specific therapeutic modalities individually, namely for ICIs (avelumab + axitinib) vs. TKIs (sunitinib), respectively. Additionally, significant associations were also seen between PFS rates and mutational profiles, as well as histocompatibility leukocyte antigen variability, whereas PD-L1 expression and tumor mutational burden did not demonstrate any predictive value [232].

In a conceptually similar manner but targeting different individual genes and evaluating a different combination of ICIs (atezolizumab + bevacizumab), the Immotion151 (NCT02420821) trial also established predictive immune response vs. angiogenesis-associated gene clusters, which showed simultaneous, yet opposing, correlations with improved PFS rates under ICIs (high immune response and/or low angiogenesis), as compared to sunitinib. Conversely, high angiogenesis signature expression associated improved PFS in the sunitinib group [233]. Corroborating these findings, the CheckMate214 trial (NCT02231749) reported that higher angiogenesis gene signature scores correlated with improved overall response rates/PFS for the sunitinib group, whereas lower angiogenesis scores associated better overall response rates in ICI group (nivolumab + ipilimumab) [215]. Moreover, high expression of inflammatory response activation and mesenchymal transition pathognomonic gene clusters was frequently encountered in patients with prolonged PFS (>18 months) [178].

Hopefully, further investigations will be able to validate existing data and provide much needed clarity regarding the role of biomarkers in RCC treatment response assessment, ultimately allowing for the elaboration and standardization of a formal and integrative, clinical/molecular case selection protocol.

## 6. Conclusions

In hindsight, the past five decades have seen significant developments in RCC detection and clinical evaluation modalities, as well as paradigm-shifting discoveries regarding RCC molecular pathology and RCC-driving metabolomics. Clinically, these developments have already translated into greatly improved detection capacities for occult RCCs, increasingly accurate and reliable RCC clinical staging protocols based on multimodal imaging evaluation, and more comprehensive RCC clinical definitions. Molecular insights into RCC carcinogenesis and proliferation have greatly nuanced contemporary clinical guidelines for the pathological evaluation of RCCs, providing better substantiated RCC differential diagnosis and subtyping protocols, and more objective risk stratification models. Notwithstanding the advent of RCC-associated-antigen targeted systemic therapy, nor the promising developments seen within the emerging, novel RCC immunotherapy clinical applications, advanced/systemically disseminated RCC remains incurable, with currently available therapeutic modalities only being able to prolong survival. Confoundingly, overall RCC mortality rates have been steadily rising for decades, in small but constant yearly increments, seemingly uninfluenced by the vast improvements made in RCC clinical management. Thus, although increasingly complex, currently available RCC clinical tools for prognosis assessment, treatment response prediction and systemic therapy are still seemingly insufficient for achieving true RCC curability and require urgent further improvement. The pressing and long-standing need for adequate RCC biomarkers, to resolve these deficits, remains unaddressed. The vast amount of existing data, regarding individual, promising RCC biomarkers must undergo extensive reevaluation and validation by external investigators in order to establish reproducibility, clearly define clinical utility, and standardize reporting and technical methodology. These are crucial conditions for assuring the transition from fundamental research initiatives to clinical tool development.

## Data Availability

Not applicable.
